# Polyamine Oxidase Triggers H_2_O_2_-Mediated Spermidine Improved Oxidative Stress Tolerance of Tomato Seedlings Subjected to Saline-Alkaline Stress

**DOI:** 10.3390/ijms23031625

**Published:** 2022-01-30

**Authors:** Jianyu Yang, Pengju Wang, Suzhi Li, Tao Liu, Xiaohui Hu

**Affiliations:** 1 College of Horticulture, Northwest A&F University, Yangling, Xianyang 712100, China; 2017060141@nwafu.edu.cn (J.Y.); wangpengju1998@nwafu.edu.cn (P.W.); 1249748926@nwafu.edu.cn (S.L.); 2 College of Horticulture, Shenyang Agricultural University, Shenyang 110866, China; 3 Key Laboratory of Protected Horticultural Engineering in Northwest, Ministry of Agriculture, Yangling, Xianyang 712100, China; 4 Shaanxi Protected Agriculture Research Centre, Yangling, Xianyang 712100, China

**Keywords:** spermidine, hydrogen peroxide, polyamine oxidase, saline-alkaline stress, *Solanum lycopersicum*

## Abstract

Saline-alkaline stress is one of several major abiotic stresses in crop production. Exogenous spermidine (Spd) can effectively increase tomato saline-alkaline stress resistance by relieving membrane lipid peroxidation damage. However, the mechanism through which exogenous Spd pre-treatment triggers the tomato antioxidant system to resist saline-alkaline stress remains unclear. Whether H_2_O_2_ and polyamine oxidase (PAO) are involved in Spd-induced tomato saline-alkaline stress tolerance needs to be determined. Here, we investigated the role of PAO and H_2_O_2_ in exogenous Spd-induced tolerance of tomato to saline-alkaline stress. Results showed that Spd application increased the expression and activities of superoxide dismutase (SOD), catalase (CAT), ascorbate peroxidase (APX), glutathione reductase (GR), and the ratio of reduced ascorbate (AsA) and glutathione (GSH) contents under saline-alkaline stress condition. Exogenous Spd treatment triggered endogenous H_2_O_2_ levels, *SlPAO4* gene expression, as well as PAO activity under normal conditions. Inhibiting endogenous PAO activity by 1,8-diaminooctane (1,8-DO, an inhibitor of polyamine oxidase) significantly reduced H_2_O_2_ levels in the later stage. Moreover, inhibiting endogenous PAO or silencing the *SlPAO4* gene increased the peroxidation damage of tomato leaves under saline-alkaline stress. These findings indicated that exogenous Spd treatment stimulated *SlPAO4* gene expression and increased PAO activity, which mediated the elevation of H_2_O_2_ level under normal conditions. Consequently, the downstream antioxidant system was activated to eliminate excessive ROS accumulation and relieve membrane lipid peroxidation damage and growth inhibition under saline-alkaline stress. In conclusion, PAO triggered H_2_O_2_-mediated Spd-induced increase in the tolerance of tomato to saline-alkaline stress.

## 1. Introduction

As sessile organisms, plants regularly face a variety of abiotic stresses, including salt [1], drought [2], and extreme temperatures stress [3] throughout their life; these stresses seriously affect their growth, development, and productivity [4,5]. Soil salinization and alkalization are widespread environmental problems, and saline-alkaline stress is more destructive than neutral salt and other abiotic stresses [6,7]. Saline-alkaline stress-induced osmotic stress and ion toxicity result in metabolic disorders, increased electrolyte leakage, cell membrane permeability, excessive accumulation of reactive oxygen species (ROS), DNA damage, protein degradation, and inhibition of plant growth and development [8,9]. Plants perceive and defend against saline-alkaline stress through complex signal transduction pathways [10,11] to activate molecular, physiological, and biochemical responses, such as accumulating low molecular weight osmolytes (proline and PAs) [12], regulation of ion absorption and homeostasis [11,13], and activation of an antioxidant system to maintain internal redox homeostasis [2,12].

H_2_O_2_ is the most abundant and relatively stable ROS in plant cells; it regulates redox signaling and metabolic pathways in response to salinity stress [2]. H_2_O_2_ is primarily formed in chloroplasts, mitochondria, peroxisomes, cytosol, and apoplast, which are mediated by NADPH oxidases (RBOH), diamine, and apoplastic polyamine oxidases (PAO) metabolic pathways, or external sources, etc. [14]. They are then removed or detoxified by an array of antioxidative enzymes and antioxidants [14]. H_2_O_2_ acts as a double-edged sword in plant stress response [14,15]. H_2_O_2_ accumulated in plant cytoplasmic exosomes acts as a signal molecule to respond to saline-alkaline or other abiotic stresses within a short period of time [4,12,16,17] and then activates downstream signal molecules (such as NO) [18] or kinases (MPK1/2) [19], and thereby ultimately triggers the plant’s defense system. However, plants under long-term or severe saline-alkaline stress accumulated excessive H_2_O_2,_ finally causing membrane lipid peroxidation damage, cell structure deformation or degradation, plant metabolism disorder, and inhibition of plant growth and development [12,20,21].

Polyamines (PAs), such as putrescine (Put), spermidine (Spd), and spermine (Spm), are low-molecular-weight aliphatic polyanionic amines that are ubiquitous in all living organisms [12,22,23]. PAs may trigger downstream signal molecules (such as H_2_O_2_ and nitric oxide); they are involved in plant response and resistance to various stresses [1,24,25] and maintain plant ion balance and redox homeostasis [26]. PA oxidation functions in the signal transduction process of plants during biotic and abiotic stress responses [27]. PAO is a key enzyme of polyamine catabolism that catalyzes the oxidation of Put or higher polyamines (Spd and Spm) and generates H_2_O_2_ [28,29]. Seven *SlPAO* genes are found in tomatoes, but only *SlPAO4* is highly conserved to *AtPAO4* and responds to exogenous PAs application and to low temperature, salt, and drought stresses [30]. PA oxidation triggers H_2_O_2_, which plays a role in the signal transduction process of plants during biotic and abiotic stress responses [27]. PAO catalyzes polyamine oxidation but also back-conversion, generating H_2_O_2_ which signals various downstream responses related to growth, development, and stresses [31,32,33,34,35,36], and stomata closure [37]. The apoplast H_2_O_2,_ also generated by NADPH oxidase encoded by respiratory burst oxidase homologs (*RBOH*s), is implicated in plants’ responses to abiotic stress [38,39]. NADPH oxidase and PAO may form a nexus and cross-talk in the frame of the strategy of plant cells to regulate ROS homeostasis [31]. Our previous studies revealed that exogenous Spd could enhance tomato’s saline-alkaline tolerance by relieving membrane lipid peroxidation damage and regulating photosynthetic capacity [20,22]. However, how exogenous Spd pre-treatment triggers the antioxidant system of tomato seedlings to resist saline-alkaline stress is still unclear. Whether PAO/NADPH oxidase triggered H_2_O_2_ is related to the roles of Spd needs to be determined. Therefore, in this study, we tried to clarify the antioxidant effect of exogenous Spd on tomatoes under saline-alkaline stress and determine whether PAO/NADPH oxidase-induced H_2_O_2_ participates in this regulatory pathway.

## 2. Results

### 2.1. Exogenous Spd Pretreatment Improved Tomato Seedling Growth under Saline-Alkaline Stress

Exogenous Spd pre-treatment had no significant effect on plants’ growth under normal conditions compared with control plants (Table 1, Figure S1). Saline-alkaline stress severely decreased plant height, stem diameter, fresh weight, dry weight, and SI by 27.6%, 13.0%, 38.8%, 38.7%, and 25.1%, respectively, compared with control plants. Spd pre-treatment plus saline-alkaline stress treatment increased plants height, stem diameter, fresh weight, dry weight, and SI by 11.0%, 12.7%, 24.7%, 23.5%, and 12.7%, respectively, compared with saline-alkaline stressed plants.

### 2.2. Exogenous Spd Pretreatment Alleviated the Oxidative Damage of Tomato Seedlings under Saline-Alkaline Stress

**Figure 1:**
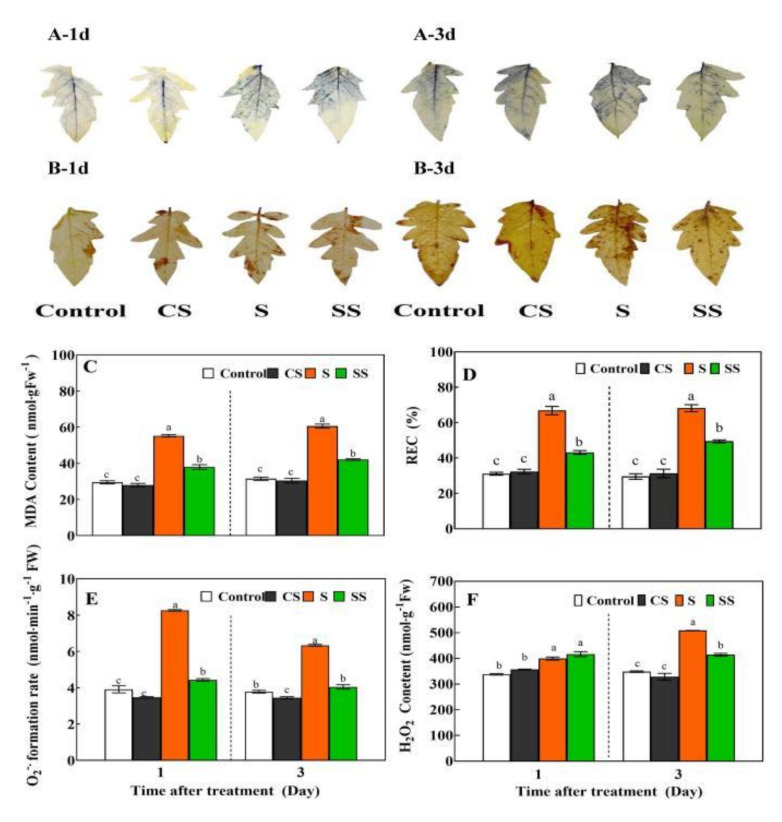
Effects of Spd pre-treatment on REC, MDA content, O_2_^−^ and H_2_O_2_ levels in tomato leaves under saline-alkaline stress. Seedlings were pretreated with 5 mL distilled water or 0.25 mM Spd and cultivated under normal conditions for 24 h, and then seedlings were irrigated with 100 mL of half strength Hoagland nutrient solution or 100 mL 300 mM saline-alkaline mixed solution. Saline-alkaline mixed solution (1:9:9:1 molar ratio of NaCl:Na_2_SO_4_:NaHCO_3_:Na_2_CO_3_, Hu et al., 2014) was added to half-strength Hoagland’s nutrient solution to a final concentration of 300 mM (pH 8.6 ± 0.2). Control, pre-sprayed distilled water under normal conditions; CS, 0.25 mM Spd foliar pre-spraying under normal conditions; S, irrigation with saline-alkaline mixed solution and H_2_O foliar pre-spraying; SS, 0.25 mM Spd foliar pre-spraying under salinity-alkalinity stress. The histochemical staining with NBT and DAB for detection of O_2_^−^ (**A**) and H_2_O_2_ (**B**); MDA content (**C**); REC (**D**); O_2_^−^ generation rate (**E**) and H_2_O_2_ content (**F**) were measured using fifth leaves of tomato seedlings after saline-alkaline stressed for 1 and 3 days. Data are expressed as the mean ± standard error of three independent biological replicates. Different letters indicate significant differences of *p* < 0.05 according to Tukey’s test.

Excessive ROS accumulation in plants caused membrane lipid peroxidation and increased electrolyte leakage. Pre-spraying with Spd had no significant effects on REC, MDA content, O_2_^−^ generation rate, and H_2_O_2_ content in tomato leaves, compared with control plants under normal conditions (Figure 1). Compared with control plants, saline-alkaline stress increased REC, MDA content, O_2_^−^ generation rate, and H_2_O_2_ content of tomato leaves after being subjected to stress for 1 and 3 days. Under saline-alkaline stress, Spd pretreated plants showed reduced REC, O_2_^−^ generation rate, and MDA content compared with untreated plants after 1 day (by 35.5%, 27.5%, and 46.4%) and 3 days (by 36.1%, 31.2%, and 30.06%, respectively).

Spd plus saline-alkaline stress treatment resulted in the significant reduction of the H_2_O_2_ content of tomato leaves compared with plants subjected to saline-alkaline treatment alone after being stressed for 3 days. However, no significant difference was found when compared with plants subjected to stress for 1 day. Histochemical staining results were consistent with the content determination findings (Figure 1A,B).

### 2.3. Effects of Spd Pre-Spraying on Antioxidation in Tomato Plants under Saline-Alkaline Stress

**Figure 2:**
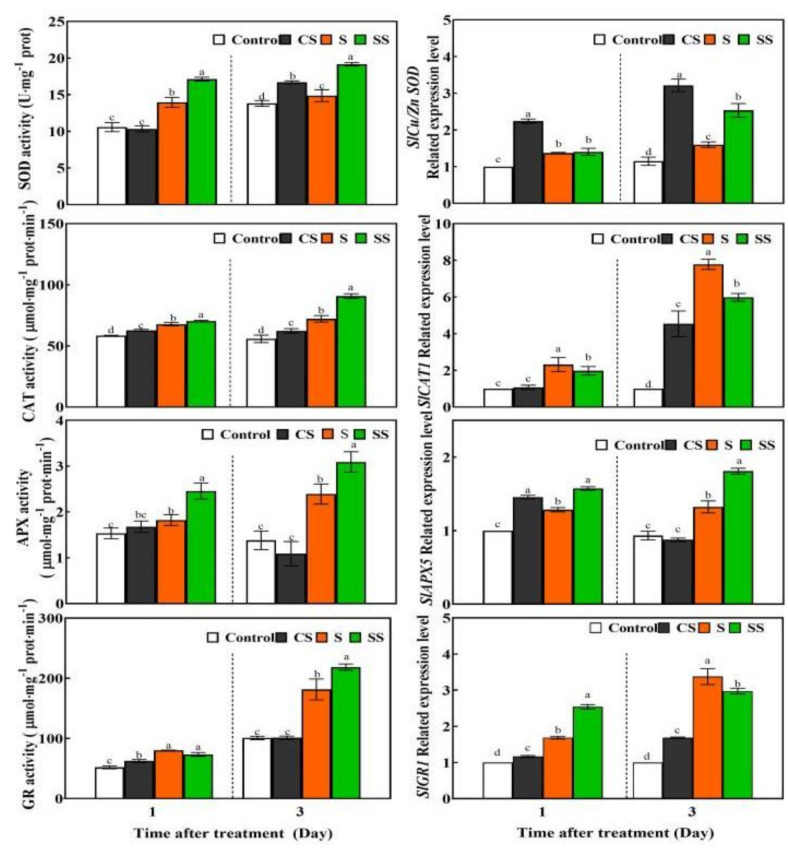
Effects of Spd pre-treatment on activities of antioxidase and the expression of related genes in tomato leaves under saline-alkaline stress. Seedlings were pretreated with 5 mL distilled water or 0.25 mM Spd and cultivated under normal conditions for 24 h, and then seedlings were irrigated with 100 mL of half strength Hoagland nutrient solution or 100 mL 300 mM saline-alkaline mixed solution. Saline-alkaline mixed solution (1:9:9:1 molar ratio of NaCl:Na_2_SO_4_:NaHCO_3_:Na_2_CO_3_, Hu et al., 2014) was added to half-strength Hoagland’s nutrient solution to a final concentration of 300 mM (pH 8.6 ± 0.2). Control, pre-sprayed distilled water under normal conditions; CS, 0.25 mM Spd foliar pre-spraying under normal conditions; S, irrigation with saline-alkaline mixed solution and H_2_O foliar pre-spraying; SS, 0.25 mM Spd foliar pre-spraying under salinity-alkalinity stress. The activities of SOD, CAT, APX, GR and the expression of related genes in fifth leaf of seedlings were measured after saline-alkaline stressed for 1 and 3 days. Data are expressed as the mean ± standard error of three independent biological replicates. Different letters indicate significant differences of *p* < 0.05 according to Tukey’s test.

**Figure 3:**
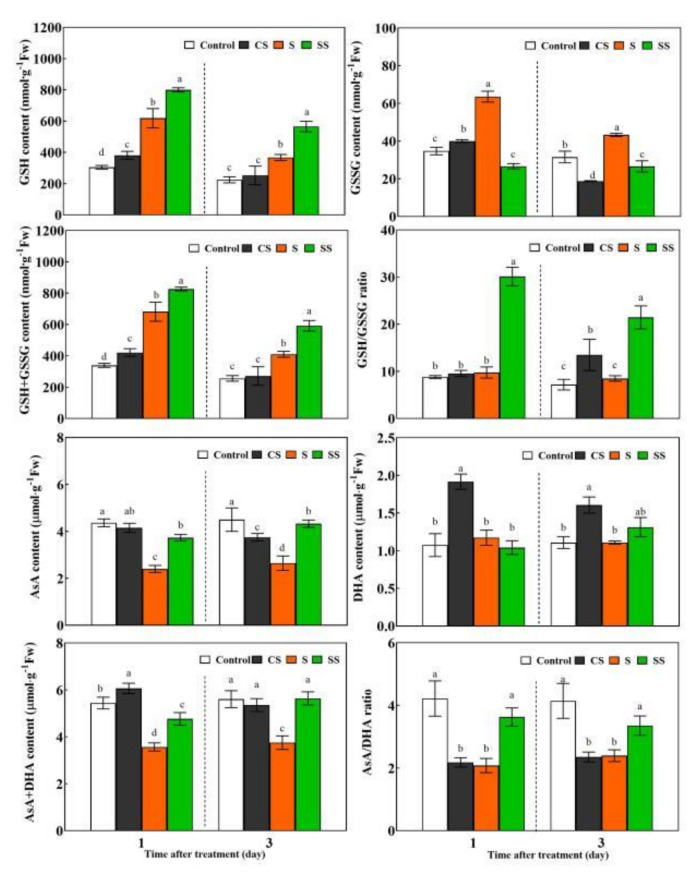
Effects of Spd pre-treatment on the redox status of glutathione and ascorbate in tomato leaves under saline-alkaline stress. Seedlings were pretreated with 5 mL distilled water or 0.25 mM Spd and cultivated under normal conditions for 24 h, then seedlings were irrigated with 100 mL a half strength Hoagland nutrient solution or 100 mL 300 mM saline-alkaline mixed solution. Saline-alkaline mixed solution (1:9:9:1 molar ratio of NaCl:Na_2_SO_4_:NaHCO_3_:Na_2_CO_3_, Hu et al., 2014) was added to half-strength Hoagland’s nutrient solution to a final concentration of 300 mM (pH 8.6 ± 0.2). Control, pre-sprayed distilled water under normal conditions; CS, 0.25 mM Spd foliar pre-spraying under normal conditions; S, irrigation with saline-alkaline mixed solution and H_2_O foliar pre-spraying; SS, 0.25 mM Spd foliar pre-spraying under salinity-alkalinity stress. The GSH, GSSG, AsA and DHA content in fifth leaves of seedlings were measured after saline-alkaline stressed for 1 and 3 days. Data are expressed as the mean ± standard error of three independent biological replicates. Different letters indicate significant differences of *p* < 0.05 according to Tukey’s test.

Spd pre-treatment could increase the activities of SOD, CAT, and GR, the gene expression of *SlCu/ZnSOD*, *SlCAT1*, *SlAPX5,* and *SlGR1*, as well as the contents of GSH, GSH + GSSG, GSH/GSSG, DHA, and AsA + DHA of plants under normal conditions for 1 day and/or 3 days (Figure 2 and Figure 3).

Saline-alkaline stress increased the enzyme activities of SOD, CAT, APX, GR, the gene expression of *SlCu/ZnSOD*, *SlCAT1*, *SlAPX5* and *SlGR1*, as well as the content of GSH, GSSG, and GSH + GSSG, but reduced the content of AsA, AsA + DHA and AsA/DHA compared with control plants. Spd plus saline-alkaline stress treated plants showed increased activities and gene expressions of SOD, CAT, APX, and GR, as well as the elevation of the reduced GSH and AsA contents and ratio, compared with seedlings subjected to saline-alkaline stress without Spd pre-treatment for 1 and 3 days (Figure 2 and Figure 3).

Spd pre-treatment significantly increased the PAO activity (at 1 day) and *SlPAO4* gene expression (at 1 and 3 days), compared to control plants under normal conditions (Figure S2). Saline-alkaline stress increased the PAO activity and *SlPAO4* gene expression at 1 and 3 days, compared to control plants. While Spd plus saline-alkaline stress treated plants dramatically enhanced PAO activity and *SlPAO4* gene expression at 1 day, but significantly reduced them at 3 days, compared to saline-alkaline stressed plants alone (Figure S2).

### 2.4. PAO Triggered H_2_O_2_ Accumulation Was Involved in Spd’s Alleviation of Oxidative Stress Damage of Tomato Leaves under Normal or Saline-Alkaline Stress Conditions

**Figure 4:**
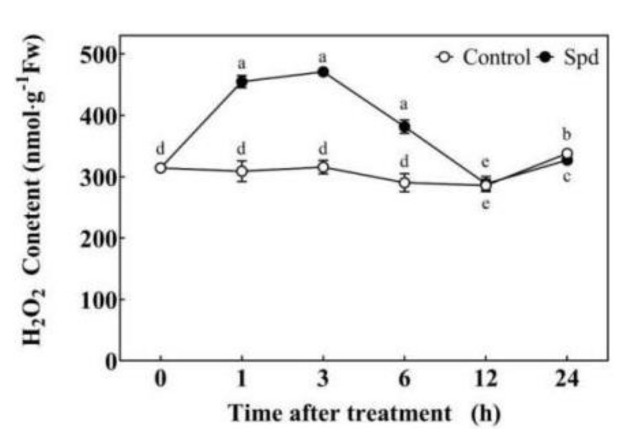
The dynamic change of H_2_O_2_ content in Spd treated tomato leaves under normal conditions. The control and Spd-treated seedlings were sprayed with 5 mL distilled water (Control) or 0.25 mM Spd (Spd) cultivated under normal conditions for 24 h. The fifth leaves of tomato seedlings were harvested after Spd spraying for 0, 1, 3, 6, 12, 24 h. Data are expressed as the mean ± standard error of three independent biological replicates. Different letters indicate significant differences of *p* < 0.05 according to Tukey’s test.

**Figure 5:**
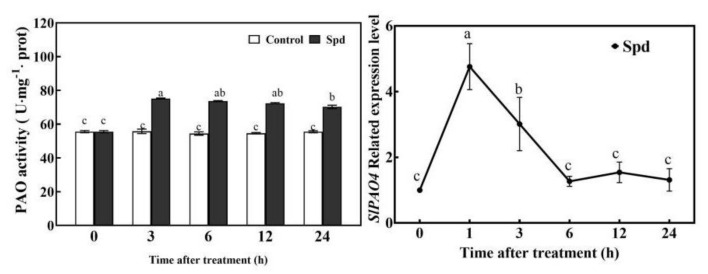
Effects of Spd pre-spraying on dynamic change of PAO activities and *SlPAO4* gene expression in tomato leaves under normal conditions. Seedlings were sprayed with 5 mL distilled water (Control) or 0.25 mM Spd (Spd) cultivated under normal conditions for 24 h. The fifth leaves of tomato seedlings were harvested after Spd spraying for 0, 1, 3, 6, 12, 24 h. Data are expressed as the mean ± standard error of three independent biological replicates. Different letters indicate significant differences of *p* < 0.05 according to Tukey’s test.

The H_2_O_2_ content gradually increased and then decreased after Spd treatment in tomato leaves under normal conditions. H_2_O_2_ content peaked at 3 h (Figure 4). Exogenous Spd treatment significantly enhanced the activity of PAO and the expression of *SlPAO4*. The PAO activity peaked at 3 h, whereas *SlPAO4* expression peaked at 1 h by 3.69-fold, compared with control plants (Figure 5). The expressions of other *SlPAO* genes decreased (*SlPAO1*, *5*, and *6*), or increased (*SlPAO2* and *7*) at a later stage, showing just a small increase (*SlPAO3*) in Spd-pretreated plants (Figure S3). Exogenous Spd did not up-regulate *SlRBOH1* gene expression under normal conditions (Figures S4 and S5).

**Figure 6:**
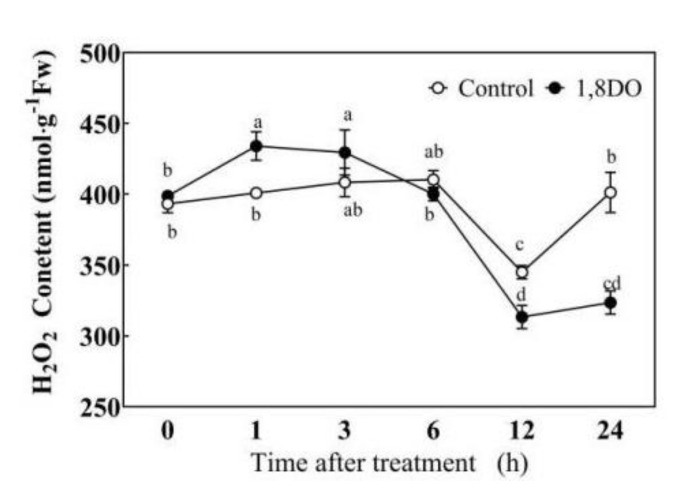
The H_2_O_2_ content of tomato seedlings treated with 5 mL distilled water (Control) or an inhibitor of PAO, 1,8-diaminooctane (1,8-DO) under normal conditions. Seedlings were sprayed with 5 mL distilled water (Control) or 1 mM 1,8-diaminooctane (1,8-DO) cultivated under normal conditions for 24 h. The fifth leaves of tomato seedlings were harvested after 1,8-diaminooctane spraying for 0, 1, 3, 6, 12, 24 h. Data are expressed as the mean ± standard error of three independent biological replicates. Different letters indicate significant differences of *p* < 0.05 according to Tukey’s test.

**Figure 7:**
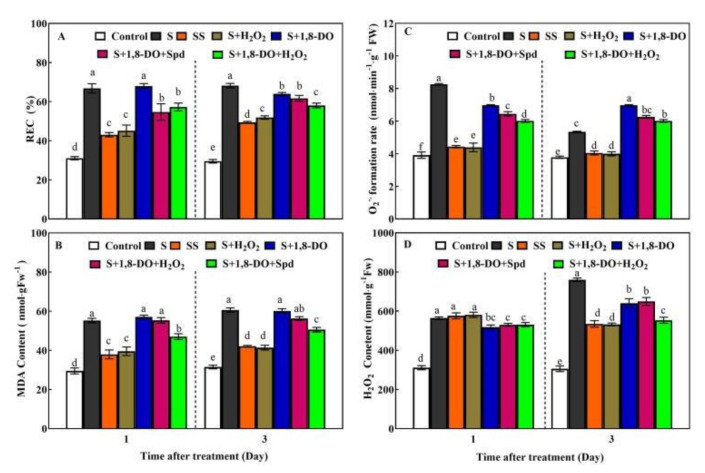
Effects of 1,8-DO on REC (**A**); MDA content (**B**); O_2_^−^ generation rate (**C**) and H_2_O_2_ content (**D**). Plants were foliar pretreated with 5 mL 1 mM 1,8-diaminooctane (1,8-DO, an inhibitor of polyamine oxidase). After 12 h, the leaves were sprayed with 5 mL distilled water, 0.25 mM Spd, or 5 mM H_2_O_2_; then seedlings were irrigated with 100 mL of half strength Hoagland nutrient solution (Control) or 100 mL 300 mM saline-alkaline mixed solution. Saline-alkaline mixed solution (molar ratio of NaCl:Na_2_SO_4_:NaHCO_3_:Na_2_CO_3_ is 1:9:9:1) was added to half-strength Hoagland’s nutrient solution to a final concentration of 300 mM (pH 8.6 ± 0.2). The fifth leaves of tomato seedlings were harvested after saline-alkaline stressed for 1 day and 3 days. Data are expressed as the mean ± standard error of three independent biological replicates. Different letters indicate significant differences of *p* < 0.05 according to Tukey’s test.

Under normal conditions, 1,8-DO was used to inhibit endogenous PAO activity, which resulted in the reduction of H_2_O_2_ level compared with control plants after 6 h; a significant reduction of H_2_O_2_ level was obtained at 12 h (Figure 6). Under saline-alkaline stress, the application of exogenous Spd or H_2_O_2_ significantly reduced the accumulation of O_2_^−^, MDA content, and REC level in tomato leaves compared with leaves from plants subjected to salt stress alone (Figure 7). Application of 1,8-DO significantly reduced the endogenous H_2_O_2_ level compared with plants subjected to saline-alkaline stress treatment alone and significantly increased the accumulation of O_2_^−^ at 3 days. Under saline-alkaline stress, 1,8-DO + Spd or 1,8-DO + H_2_O_2_ significantly increased the generation rate of O_2_^−^, the levels of MDA, and REC compared with plants subjected to the pre-treatments of Spd or H_2_O_2_ alone and only increased the endogenous H_2_O_2_ levels at 3 days.

Under saline-alkaline stress, application of Spd or H_2_O_2_ significantly increased the activities of SOD at 1 and 3 days, CAT at 1 day, APX and GR at 3 days, compared with saline-alkaline alone (Figure S6). Whereas 1,8-DO treatment significantly reduced the activities of SOD and CAT at 1 and 3 days, GR at 1 day, compared with salt stress alone. 1,8-DO + Spd treatment significantly reduced the activities of SOD, CAT, and GR at 1 and 3 days, and APX at 3 days, compared with Spd alone treatment. While 1,8-DO + H_2_O_2_ treatment significantly reduced SOD activity compared with H_2_O_2_ treatment alone at 1 day and reduced CAT, APX, and GR activities at 3 days under saline-alkaline stress condition.

### 2.5. Silencing of SlPAO4 Reduced the PAO Activity and Saline-Alkaline Stress Resistance of Tomato Seedlings

**Figure 8:**
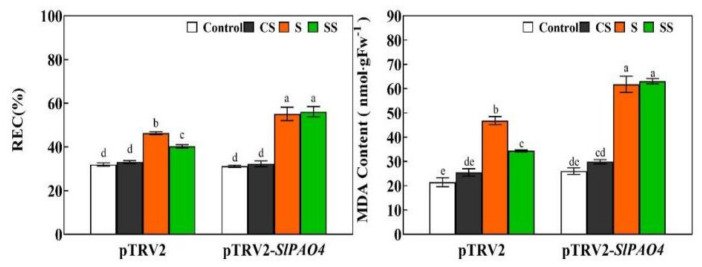
Effects of *SlPAO4* silencing on REC and MDA content in tomato leaves under saline-alkaline stress. pTRV2 and *SlPAO4* silencing tomato seedlings were pretreated with 5 mL distilled water or 0.25 mM Spd and cultivated under normal conditions for 24 h, then seedlings were irrigated with 100 mL of half strength Hoagland nutrient solution or 100 mL 300 mM saline-alkaline mixed solution. Saline-alkaline mixed solution (1:9:9:1 molar ratio of NaCl:Na_2_SO_4_:NaHCO_3_:Na_2_CO_3_, Hu et al., 2014) was added to half-strength Hoagland’s nutrient solution to a final concentration of 300 mM (pH 8.6 ± 0.2). Control, pre-sprayed distilled water under normal conditions; CS, 0.25 mM Spd foliar pre-spraying under normal conditions; S, irrigation with saline-alkaline mixed solution and H_2_O foliar pre-spraying; SS, 0.25 mM Spd foliar pre-spraying under salinity-alkalinity stress. The fifth leaves of tomato seedlings were harvested after saline-alkaline stressed for 3 days. Data are expressed as the mean ± standard error of three independent biological replicates. Different letters indicate significant differences of *p* < 0.05 according to Tukey’s test.

To further prove the role of PAO in Spd induced tomato resistance to saline-alkaline stress, VIGS technology was used to silence the *SlPAO4* gene, the expression of *SlPAO4* as well as PAO activity were significantly reduced (Figure S7). Under normal conditions, spraying with or without Spd had no significant effect on the REC and MDA content in leaves of pTRV2 or pTRV2-*SlPAO4* plants (Figure 8). However, REC and MDA content in saline-alkaline stressed plants were significantly increased compared with the plants kept under normal conditions. 

Under saline-alkaline stress, the REC and MDA content in pTRV2-*SlPAO4* plants were significantly higher than those in non-silenced plants. The REC and MDA content of plants treated with saline-alkaline stress were not significantly different from those plants subjected to Spd treatment plus saline-alkaline stress in pTRV2-*SlPAO4* plants.

## 3. Discussion

During protected cultivation, crops are often subjected to saline-alkaline stress, which seriously affects normal growth, development, and yield formation. Short-term saline-alkaline stress may trigger the plant’s response and defense system, but long-term or severe stress causes the lipid peroxidation damage of the cell membrane [20,21]. Exogenous plant growth regulators are widely used to improve plant salt stress tolerance [1,12]. Our previous studies showed that exogenous Spd could enhance plant saline-alkaline stress resistance by maintaining the integrity of the chloroplast’s structure, chlorophyll synthesis, and photosynthesis to support plant growth [21,22,40]. In this study, short-term saline-alkaline stress stimulated tomato’s antioxidant system by increasing the gene expression and activities of SOD, CAT, APX, and GR as well as the GSH content, which indicates that the SOD, CAT, and AsA-GSH cycles were involved in antioxidant activity in response to saline-alkaline stress (Figure 2 and Figure 3). However, high APX activity led to an insufficient supply of its substrate AsA, which resulted in the decrease in AsA content. Ultimately, the antioxidant effect of the AsA-GSH cycle was weakened, and the excessive accumulation of ROS was not eliminated completely, thereby eventually causing cell membrane lipid peroxidation damage and plant growth inhibition (Figure 1, Table 1). Compared with salt stress, application of Spd further increased the expressions and activities of SOD, CAT, APX, GR, and the reduced ratio of AsA and GSH, which indicate that SOD, CAT, and AsA-GSH played a positive synergistic effect in eliminating the excessive accumulation of ROS, drastically reducing membrane lipid peroxidation damage. It is worth noting that these antioxidase encode by genes may be regulated at translation or post-translational levels, but not at the transcriptional levels, or genes expression occurs earlier than enzymatic activity (Figure 2). Further research is needed in the future.

ROS is a double-edged sword. Excessive ROS accumulation can cause oxidative damage, whereas moderate ROS accumulation can act as signal molecules in the cell; it responds to stress signals and transmits them to downstream signal molecules [2], such as NO [18] or kinases (MPK1/2) [19], which then activate the defense system. NADPH oxidase, which is located on the cytoplasmic membrane, and PAO, which is located on the cell wall or intracellular are two main sources of H_2_O_2_ in plants [38,40]. This study showed that exogenous Spd pre-treatment could stimulate endogenous H_2_O_2_ levels (Figure 4), *SlPAO4* gene expression as well as PAO activity (Figure 5), and inhibition of endogenous PAO activity significantly reduced H_2_O_2_ levels in the later stage under normal conditions (Figure 6). However, exogenous Spd did not trigger *SlRBOH1* gene expression (Figures S4 and S5). Thus, the following were speculated: (1). the exogenous Spd pre-treatment may trigger the H_2_O_2_ level through the action of PAO. (2). Exogenous Spd may regulate NADPH oxidase activity at post-transcriptional or translational levels, which then cooperate with PAO to regulate the production of apoplast H_2_O_2_. These still need further study.

Under saline-alkaline stress, the inhibition of PAO enzyme activity by 1,8-DO reduced the endogenous H_2_O_2_ level in tomato leaves in a short period of time (Figure 7). This may weaken the signal effects of H_2_O_2_ derived by Spd pre-treatment in responding and transmitting stress signals. Resistance may also be reduced, thereby weakening the roles of Spd and exogenous H_2_O_2_ in the alleviation of tomato saline-alkaline stress (Figure 7). Eventually, redox imbalance ensues, and excessive accumulation of O_2_^−^ and increased membrane lipid peroxidation damage occur. Compared with exogenous H_2_O_2_ and Spd treatments alone, 1,8-DO treatment further increased the level of endogenous H_2_O_2_ under saline-alkaline stress at 3 days. The following were speculated. 1. Over time, the effects of 1,8-DO may weaken in reducing H_2_O_2_ production. 2. Treatment with 1,8-DO decreased the activities of SOD, CAT, APX, and GR at 1 day (Figure S5), which could not completely remove excess H_2_O_2_ and eventually caused H_2_O_2_ accumulation at 3 days. Silencing the *SlPAO4* gene with VIGS technology further indicated that PAO might participate in the antioxidant effect of Spd in tomatoes via the H_2_O_2_ pathway (Figure 8).

## 4. Materials and Methods

### 4.1. Plant Culture and Experimental Design

Tomato (*Lycopersicon esculentum* Mill. cv. Alisa Craig) seedlings were used. The seeds were germinated at 28 °C in Petri dishes with moistened filter paper. Seedlings with fourth true leaves were transplanted into plastic pots (7 cm × 7 cm × 11 cm) filled with a mixture of peat, perlite, and vermiculite (2:1:1, *v*/*v*/*v*, pH 6.3 ± 0.1) and cultivated in a growth chamber under the following temperature, relative humidity, photoperiod, and photosynthetic photon flux density conditions: 25 °C/18 °C, 65% ± 5%, 12 h/12 h (day/night), and 350 μmol·m^−2^·s^−1^, respectively. The experiments started when the fifth true leaf was fully unfolded. Thirty tomato seedlings were used for each treatment replicate. Three biological replicates were performed for each experiment.

To determine the effects of exogenous Spd pre-spraying on tomato seedlings under saline-alkaline tolerance, four treatments were designed. (1) The seedlings were foliar pre-sprayed with 5 mL distilled water under normal conditions (irrigating with 100 mL half-strength Hoagland nutrient solution, pH 6.3 ± 0.2, Control); (2) 0.25 mM Spd (Sigma Aldrich, St. Louis, MO, USA) [22] foliar pre-sprayed under normal conditions, (CS); (3) irrigation with 100 mL 300 mM saline-alkaline mixed solution and H_2_O foliar pre-spraying, (S); (4) 0.25 mM Spd foliar pre-spraying under salinity-alkalinity stress, (irrigating with 100 mL 300 mM saline-alkaline mixed solution SS). Saline-alkaline mixed solution (1:9:9:1 molar ratio of NaCl:Na_2_SO_4_:NaHCO_3_:Na_2_CO_3_ [12] was added to half-strength Hoagland’s nutrient solution to obtain a final concentration of 300 mM (pH 8.6). The fifth leaves of tomato seedlings were harvested after saline-alkaline stressed at 1, 3, 6, 12, and 24 h to analyze the *SlRBOH1* and *SlPAO4* gene expression. Plants were treated for 1 and 3 days, after which the fifth leaves were harvested for the histochemical staining of superoxide anions (O_2_^−^) and H_2_O_2_ and to determine the contents of malondialdehyde (MDA) and H_2_O_2_, O_2_^−^ formation rate, relative electrical conductivity (REC), antioxidase (SOD, CAT, GR, and APX) and PAO activities, the content of antioxidants (GSH, GSSG, ASA, and DHA), and mRNA transcriptions of *SlCu/Zn-SOD*, *SlCAT1*, *SlRBOH1*, *SlPAO4*, *SlAPX5*, *SlGR1*, and *SlPAO4*. The growth indexes were measured after being stressed for 6 days, and the plants’ phenotype was taken photos after the plants were subjected to saline-alkaline stress for 3 days.

To study the effects of Spd on the dynamic change of H_2_O_2_ content, PAO activity, and *SlPAO1-7* and *SlRBOH1* gene expression after Spd treatment, the plants were treated with distilled water (control) or 0.25 mM Spd under normal conditions. PAO activities, the levels of H_2_O_2_ content, and the transcripts of *SlPAO1-7* and *SlRBOH1* were measured after Spd treatment at 1, 3, 6, 12, and 24 h.

To determine the role of PAO in Spd-induced oxidative stress tolerance under saline-alkaline stress, the plants were foliar pre-treatment with 1 mM 1,8-diaminooctane (1,8-DO), which is an inhibitor of polyamine oxidase [32]. After 12 h, the leaves were sprayed with 0.25 mM Spd or 5 mM H_2_O_2_ [3]. After 24 h, plants were subjected to 300 mM saline-alkaline stress. The fifth leaf of plants was collected at 1 and 3 days after saline-alkaline stress to measure the activities of antioxidant enzyme (SOD, CAT, APX, and GR), the MDA content, H_2_O_2_ content, O_2_^-^ formation rate, and REC.

To explore the function of *SlPAO4* in Spd-induced saline-alkaline tolerance, we pretreated the pTRV2 and *SlPAO4* silenced (pTRV2-*SlPAO4*) tomato seedlings with distilled water or 0.25 mM Spd. These tomato seedlings were grown under normal conditions for 24 h, after which they were subjected or not subjected to salinity-alkalinity stress. After 3 days, the fifth leaves were harvested to analyze the degree of stress tolerance by measuring the changes in MDA content and REC.

### 4.2. Construction of Virus-Mediated Gene-Silencing Vector

We obtained the cDNA sequence of tomato *SlPAO4* from the Solanaceae database (https://www.sgn.cornell, accessed on 4 May 2021). The 300 bp fragments of the *SlPAO4* gene were PCR amplified by using primers (forward primer: GTGAGAAGGTTACCGAATCTCTTGCTTGTGACCTCG AGAAATT, reversal primer: CGTGAGCTCGGTACCGGATCCACGTTTCACCAGCCATA ATTCC), which contained EcoR I and BamH I restriction enzyme site. The target sequence was constructed on the pTRV2 vector via homologous recombination. The constructed vector was transformed into agrobacterium tumefaciens *GV3101*, and then the expanded cotyledon leaves of tomatoes were infected into a mixed culture of agrobacterium tumefaciens containing the pTRV1:pTRV2-*SlPAO4* (1:1, *v*/*v*) [18]. Tomato seedlings infected into *A. tumefaciens* containing pTRV1: pTRV2 (1:1, *v*/*v*) were regarded as the control. The plants were grown at 20 °C for 3 days before used in experiments, which can improve the gene-silencing efficiency [41]. After this period, all seedlings were cultivated in a growth chamber in which the temperature, relative humidity, photoperiod, and photosynthetic photon flux density were 25/18 °C, 65 ± 5%, 12/12 h (day/night), 350 μmol·m^−2^·s^−1^, respectively. The *SlPAO4*-silencing efficiency was determined by the *SlPAO4* relative mRNA expression of each plant infected with *A. tumefaciens* containing pTRV1: pTRV2-*SlPAO4* from the fifth leaves of tomato. The expression of *SlPAO4* in plants infected with *A. tumefaciens* containing pTRV1: pTRV2 was normalized as 1. The plants with *SlPAO4* expression levels 60% lower were selected as *SlPAO4* silencing tomato seedlings.

### 4.3. Determination of Plant Growth

Five uniform seedlings were detached by uprooting. The fresh weight (FW) and dry weight (DW) of whole plants were determined. The plants were washed with distilled water, and the water was absorbed with absorbent paper, then the FW was measured. DW was determined after drying for 15 min at 105 °C and then oven-drying at 75 °C until a constant weight was obtained. The plant height was measured by using a ruler, and the stem diameter was measured by using a vernier caliper. Seedling index (SI) was calculated according to the method of Xu et al. [11].

### 4.4. Analysis of Plants Lipid Peroxidation

The injury level of lipid peroxidation in leaves was assessed by measuring the MDA content using the method described by Hodges et al. [42]. REC was determined and calculated according to the method described by Zhou and Leul [43].

### 4.5. Analysis of ROS

The analysis of O_2_^−^ generation rate and H_2_O_2_ content. The O_2_^−^ generation rate was determined by using the method of Elstner and Heupel [44]. H_2_O_2_ content was measured by using the method described by Su [45]. The histochemical staining of O_2_^−^ and H_2_O_2_ was performed according to the methods of Jabs [46] and Thordal-Christensen [47].

### 4.6. Measurement of Antioxidant Enzyme Activity, Antioxidant Contents, and PAO Activity

The activity SOD (EC 1.15.1.1) was assayed by using the method of Stewart and Bewley [48]. CAT (EC 1.11.1.6), GR (EC 1.6.4.2), and APX (EC 1.11.1.11) activities were determined as described by Noctor et al. [49]. The antioxidant contents of GSH, GSSG, AsA, and DHA were measured according to the methods of Noctor et al. [49]. The PAO activity in the roots and leaves was measured according to the methods of Mhaske et al. [50].

### 4.7. Analysis of Gene Expression

Gene expression was measured by performing real-time quantitative PCR. Total RNA was extracted using a Plant RNA Extraction Kit (Omega Bio-Tek, Doraville, GA, USA) according to the manufacturer’s protocol. RNA was reverse transcribed using the Prime Script^TM^ RT Reagent Kit with a gDNA Eraser (Takara, Shiga, Japan) according to the manufacturer’s protocol. Actin7 was used as an internal control. The relative level of gene expression was calculated according to Livak and Schmittgen [51]. The gene-specific primers of *SlPAO1~7*, *SlRBOH1, SlCu/Zn-SOD*, *SlCAT1*, *SlAPX5*, and *SlGR1* are listed in Supplementary Table S1.

### 4.8. Statistical Analysis

All data were analyzed with SPSS 20 software (IBM) using Tukey’s multiple range test at a significance level of *p* < 0.05 unless stated otherwise. Each reported data point represented the average of three biological replicates (*n* = 3) unless stated otherwise, and each experiment was repeated thrice.

## 5. Conclusions

Exogenous Spd pre-treatment stimulated the expressions of *SlPAO* genes (*SlPAO4*) and increased PAO enzyme activity, which then elevated H_2_O_2_ level, improved plant response to saline-alkaline stress signals, and activated the downstream antioxidant system to eliminate excessive ROS accumulation and relieve membrane lipid peroxidation damage and growth inhibition.

## Figures and Tables

**Table 1:** Exogenous Spd pretreatment improved tomato seedlings growth under saline-alkaline stress.

Treatment	Plant Height (cm)	Stem Diameter (mm)	Fresh Weight (g)	Dry Weight (g)	Seedling Index
Control	25.77 ± 0.73 ^a^	5.52 ± 0.37 ^ab^	14.29 ± 0.78 ^a^	1.11 ± 0.07 ^a^	6.41 ± 0.42 ^ab^
CS	26.83 ± 0.81 ^a^	6.30 ± 0.12 ^a^	14.09 ± 1.146 ^ab^	1.15 ± 0.03 ^a^	7.30 ± 0.68 ^a^
S	18.67 ± 0.49 ^b^	4.80 ± 0.03 ^b^	8.74 ± 0.41 ^c^	0.68 ± 0.04 ^c^	4.67 ± 0.43 ^b^
SS	20.73 ± 0.84 ^b^	5.41 ± 0.22 ^ab^	10.90 ± 0.39 ^bc^	0.84 ± 0.04 ^b^	5.26 ± 0.24 ^ab^

Note: Seedlings were pretreated with 5 mL distilled water or 0.25 mM Spd and cultivated under normal conditions for 24 h, then seedlings were irrigated with 100 mL of half strength Hoagland nutrient solution or 100 mL 300 mM saline-alkaline mixed solution. Saline-alkaline mixed solution (1:9:9:1 molar ratio of NaCl:Na_2_SO_4_:NaHCO_3_:Na_2_CO_3_, Hu et al., 2014) was added to half-strength Hoagland’s nutrient solution to a final concentration of 300 mM (pH 8.6 ± 0.2). Control, presprayed distilled water under normal conditions; CS, 0.25 mM Spd foliar pre-spraying under normal conditions; S, irrigation with saline-alkaline mixed solution and H_2_O foliar pre-spraying; SS, 0.25 mM Spd foliar pre-spraying under salinity-alkalinity stress. The plant growth indexes were measured after saline-alkaline stressed for 6 days. Data are expressed as the mean ± standard error of five independent biological replicates. Different letters indicate significant differences of *p* < 0.05 according to Tukey’s test.

## Data Availability

Not applicable.

## Supplementary Materials

The following supporting information can be downloaded at: https://www.mdpi.com/article/10.3390/ijms23031625/s1.

## Abbreviations

APX, ascorbate peroxidase; CAT, catalase; 1,8-DO, 1,8-diaminooctane; PAO, polyamine oxidase; PAs, polyamines; Put, putrescine; Spm, spermine; Spd, spermidine; Oabcdeedcba, superoxide anions; H_2_O_2_, hydrogen peroxide; REC, relative electrical conductivity; SOD, superoxide dismutase; REC, relative electrical conductivity; GSH, reduced glutathione; GSSG, oxidized glutathione; AsA, ascorbate; DHA, dehydroascorbic acid; GR, glutathione reductase; SI, seedling index.
