# Supplementary material for: Polyamine Oxidase Triggers H2O2-Mediated Spermidine Improved Oxidative Stress Tolerance of Tomato Seedlings Subjected to Saline-Alkaline Stress
Source: Int J Mol Sci. 2022 Jan 30;23(3):1625. doi: 10.3390/ijms23031625 (PMC8836047; doi:10.3390/ijms23031625)
Supplement: Supplementary file 1 [file ijms-23-01625-s001.zip › ijms-1540263-supplementary.pdf]

Supplementary files

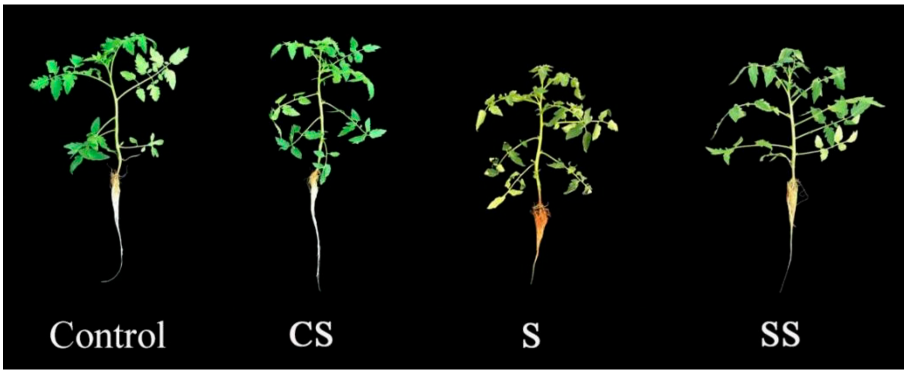

Figure S1. Effects of Spd pre-treatment on characterization of tomato plant growth under saline-alkaline stress. Seedlings were pretreated with 5 ml distilled water or 0.25 mM Spd and cultivated under normal conditions for 24 h, then seedlings were irrigated with 100 ml a half strength Hoagland nutrient solution or 100 ml 300 mM saline-alkaline mixed solution. Saline-alkaline mixed solution (1:9:9:1 molar ratio of NaCl: Na_2SO_4: $NaHCO_3$: Na_2CO_3, Hu et al., 2014) was added to half-strength Hoagland's nutrient solution to a final concentration of 300 mM (pH 8.6±0.2). Control, pre-sprayed distilled water under normal conditions; CS, 0.25 mM Spd foliar pre-spraying under normal conditions; S, irrigation with saline-alkaline mixed solution and H_2O foliar pre-spraying; SS, 0.25 mM Spd foliar pre-spraying under salinity-alkalinity stress. After 3 d of stress, the plants were taken photos.

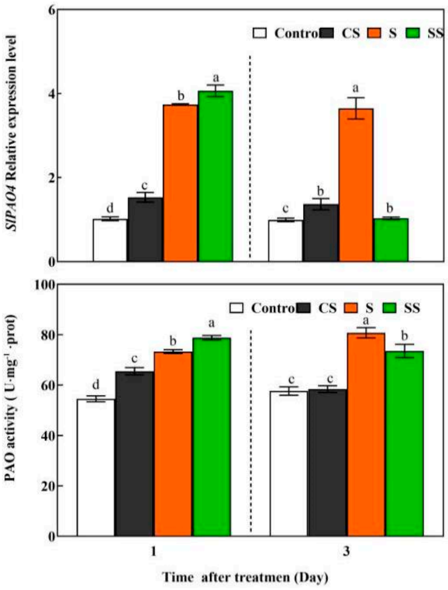

Figure S2. Effects of Spd pre-treatment on activity of PAO and the expression of *SlPAO4* gene in tomato leaves under saline-alkaline stress. Seedlings were pretreated with 5 ml distilled water or 0.25 mM Spd and cultivated under normal conditions for 24 h, then seedlings were irrigated with 100 ml a half strength Hoagland nutrient solution or 100 ml 300 mM saline-alkaline mixed solution. Saline-alkaline mixed solution (1:9:9:1 molar ratio of NaCl: Na_2SO_4: $NaHCO_3$: Na_2CO_3, Hu et al., 2014) was added to half-strength Hoagland's nutrient solution to a final concentration of 300 mM (pH 8.6±0.2). Control, pre-sprayed distilled water under normal conditions; CS, 0.25 mM Spd foliar pre-spraying under normal conditions; S, irrigation with saline-alkaline mixed solution and H_2O foliar pre-spraying; SS, 0.25 mM Spd foliar pre-spraying under salinity-alkalinity stress. The activity of PAO and the expression of *SlPAO4* gene in fifth leave of seedlings were measured after saline-alkaline stressed for 1 and 3 days. Data are expressed as the mean ± standard error of three independent biological replicates. Different

letters indicate significant differences of *P < 0.05* according to Tukey's test.

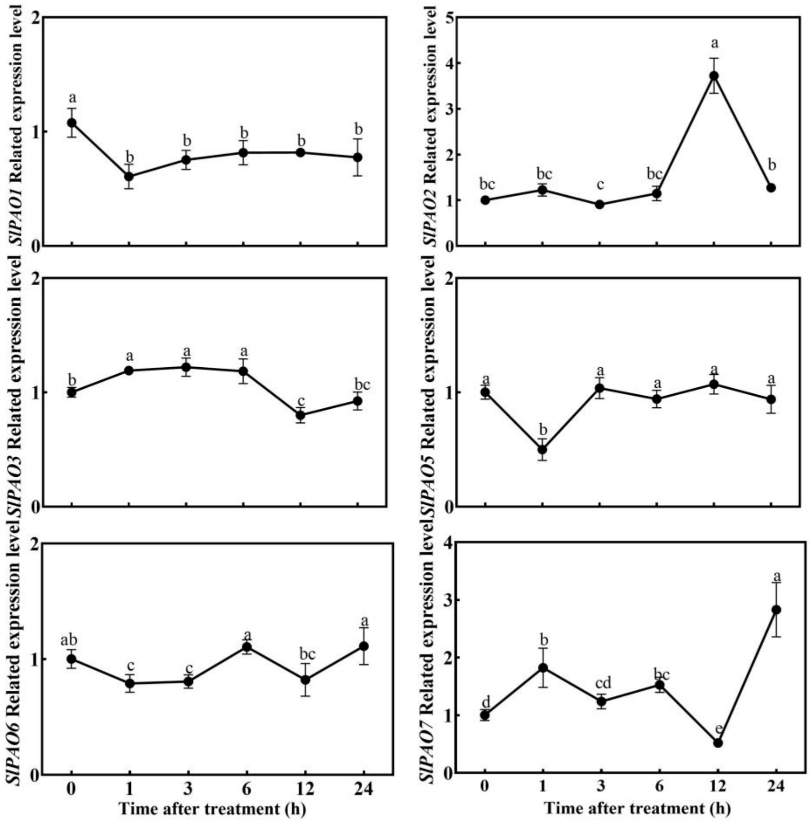

Figure S3. The *SlPAOs* genes expression level in leaves of tomato seedlings after Spd pretreatment under normal condition. The control and Spd-pretreatment seedlings were pretreated with 0.25 mM Spd (Spd) cultivated under normal conditions for 24 h. The fifth leaves of tomato seedlings were harvested after Spd spraying for 0, 1, 3, 6, 12, 24 hours. The expression of *SlPAOs* in plant at 0 h was normalized as 1. Data are expressed as the mean ± standard error of three independent biological replicates. Different letters indicate significant differences of *P < 0.05* according to Tukey's test.

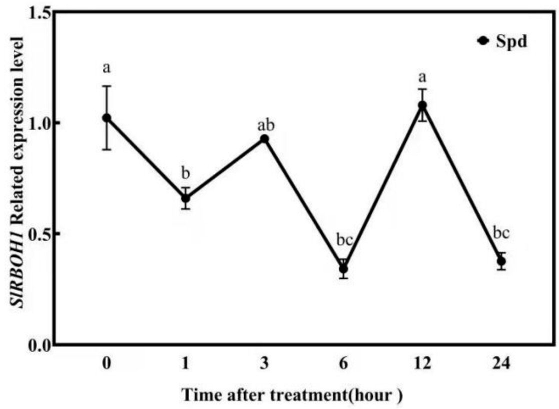

Figure S4. The *SlRBOH1* genes expression level in leaves of tomato seedlings after Spd pretreatment under normal condition. The control and Spd-pretreatment seedlings were pretreated with 0.25 mM Spd (Spd) cultivated under normal conditions for 24 h. The fifth leaves of tomato seedlings were harvested after Spd spraying for 0, 1, 3, 6, 12, 24 hours. The expression of *SlRBOH1* in plant at 0 h was normalized as 1. Data are expressed as the mean ± standard error of three independent biological

replicates. Different letters indicate significant differences of *P* < *0.05* according to Tukey's test.

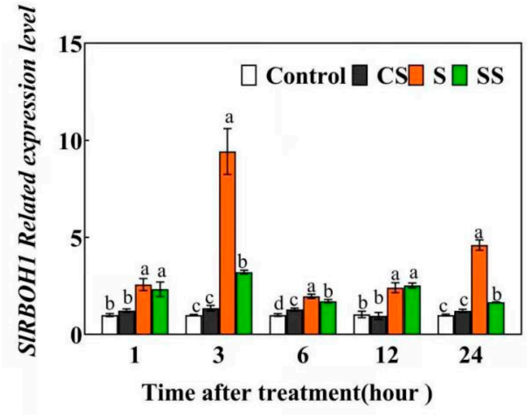

Figure S5. Effects of Spd pre-treatment on *SlRBOH1* genes expression level in leaves of tomato seedlings under saline-alkaline stress. Seedlings were pretreated with 5 ml distilled water or 0.25 mM Spd and cultivated under normal conditions for 24 h, then seedlings were irrigated with 100 ml a half strength Hoagland nutrient solution or 100 ml 300 mM saline-alkaline mixed solution. Saline-alkaline mixed solution (1:9:9:1 molar ratio of NaCl: Na_2SO_4: $NaHCO_3$: Na_2CO_3, Hu et al., 2014) was added to half-strength Hoagland's nutrient solution to a final concentration of 300 mM (pH 8.6±0.2). Control, pre-sprayed distilled water under normal conditions; CS, 0.25 mM Spd foliar pre-spraying under normal conditions; S, irrigation with saline-alkaline mixed solution and H_2O foliar pre-spraying; SS, 0.25 mM Spd foliar pre-spraying under salinity-alkalinity stress. The fifth leaves of tomato seedlings were harvested after saline-alkaline stressed at 1, 3, 6, 12, and 24 hours. Data are expressed as the mean ± standard error of three independent biological replicates. Different letters indicate significant differences of *P* < *0.05* according to Tukey's test.

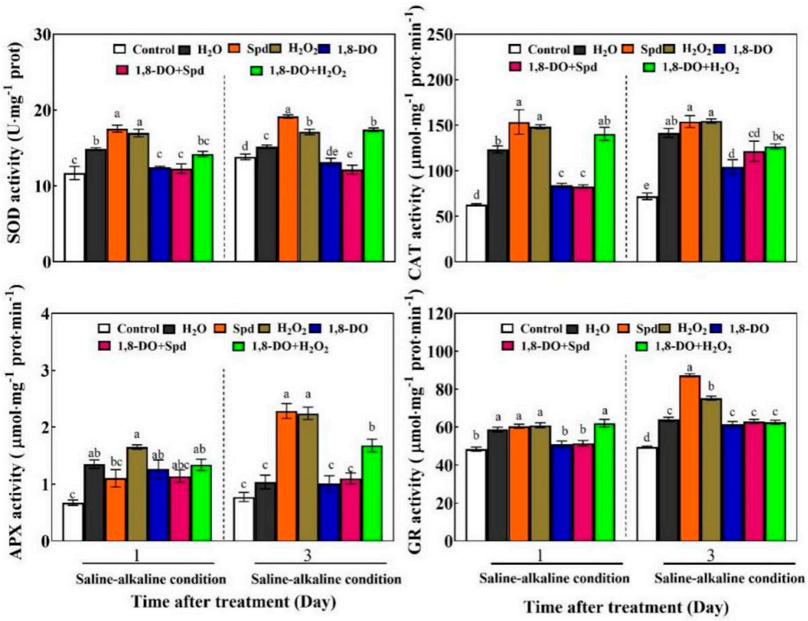

Figure S6. Effects of 1,8-DO on antioxidant enzyme activities under saline-alkaline stress. Plants were foliar pretreated with 5 ml 1 mM 1,8-diaminooctane (1,8-DO, an inhibitor of polyamine oxidase). After 12 h, the leaves were sprayed with 5ml distilled water, 0.25 mM Spd or 5 mM H_2O_2, then seedlings were irrigated with 100 ml a half strength Hoagland nutrient solution (Control) or 100 ml 300 mM

saline-alkaline mixed solution. Saline-alkaline mixed solution (molar ratio of NaCl: Na_2SO_4: $NaHCO_3$: Na_2CO_3 is 1: 9: 9: 1) was added to half-strength Hoagland's nutrient solution to a final concentration of 300 mM (pH 8.6±0.2). The fifth leaves of tomato seedlings were harvested after saline-alkaline stressed for 1 day and 3 days. Data are expressed as the mean ± standard error of three independent biological replicates. Different letters indicate significant differences of $P < 0.05$ according to Tukey's test.

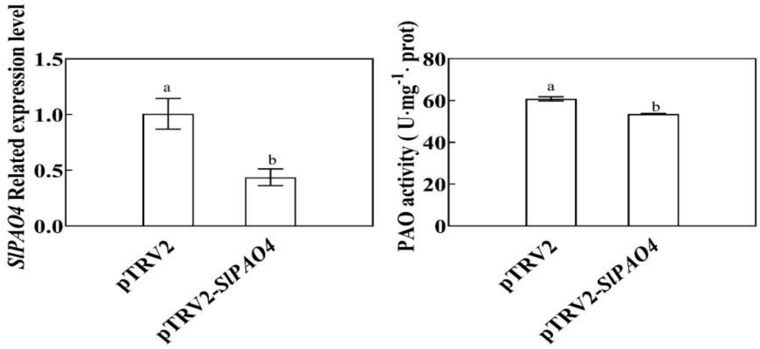

Figure S7. Expression of *SlPAO4* gene and PAO activities in pTRV2 and pTRV2-*SlPAO4* slicing plants leaves. The *SlPAO4*-silencing efficiency were determined by the *SlPAO4* expression levels of each plant infected with *A. tumefaciens* containing pTRV1: pTRV2-*SlPAO4* were determined using total RNA isolated from the fifth leaves of tomato. The expression of *SlPAO4* in plant infected with *A. tumefaciens* containing pTRV1: pTRV2 was normalized as 1. The plants with *SlPAO4* expression levels 60% lower were selected as *SlPAO4* silencing tomato seedlings. The fifth leaves of tomato were harvested before saline-alkaline stressed to determine the expression levels of *SlPAO4* gene and PAO activities. Different letters represent the significant difference at $P < 0.05$. Data are expressed as the mean ± standard error of three independent biological replicates. Different letters indicate significant differences of $P < 0.05$ according to Tukey's test.

Table S1 Gene-specific primers used for quantitative real-time PCR analysis.

Gene name	Accession numbers	Forward primer	Reverse primer
SlCu/Zn-SOD	Solyc11g066390	TCACCACAACCAGCACTACCA	AGTGACAACCCCCTCAACATTAG
SlCAT1	Solyc12g094620	CGCATACGACACCCCTTTC	CGGAGAAAATCAGCACAAGTAAG
SlAPX5	Solyc06g005160	CTGTGCTCCTATTATGCTTCGTT	AGGCGTCCCTCTTCTGGTG
SlGR1	Solyc09g091840	TCGTAATAGGTGCTGGAAGTGG	TTTTAGGAACGCAACCACGA
SlPAO1	Solyc01g087590	ATAGGCGGTAGGATAAGGAAAG	CGGAGATTAGACTGAAGAGCAA
SlPAO2	Solyc07g043590	GGGTACAGATGTAAACTCACTCG	CCTGGGTAATCCATACTCGTC
SlPAO3	Solyc12g006370	TCGCTTAGGTCACAGGGTT	GGCAGCATCGGCTACAA
SlPAO4	Solyc02g081390	ACAAGGCAACAGGACATCG	GCTTAGTGGCATCAGGGAAC
SlPAO5	Solyc03g031880	CGGAAGACTTGCTTATGACG	ATCTGTTCCCCAGCGTGA
SlPAO6	Solyc07g039310	GGAGGTAGCCCTGTTCACG	TTCCAAAAGCCCATCCATAC
SlPAO7	Solyc05g018880	GGTCTTACAGCAGCTAACAAGCT	TCCTATCACCACCCTCCACA
SlRBOH1	Solyc08g081690	CGGAACAGGCAACGGTGTA	TGCGAAATCGGAACGATAAA
actin7	Solyc03g078400	GGGATGGAGAAGTTTGGTGGTGG	CTTCGACCAAGGGATGGTGTAGC
